# Editorial: Sociomateriality in Children With Typical and/or Atypical Development

**DOI:** 10.3389/fpsyg.2020.610385

**Published:** 2020-12-10

**Authors:** Antonio Iannaccone, Federico Manzi, Monica Mollo, Giulia Savarese

**Affiliations:** ^1^Institut de Psychologie et Éducation, Université de Neuchâtel, Neuchâtel, Switzerland; ^2^Research Unit on Theory of Mind, Department of Psychology, Università Cattolica del Sacro Cuore, Milan, Italy; ^3^Department of Human, Philosophical, and Education Sciences, University of Salerno, Salerno, Italy; ^4^Department of Medicine, Surgery and Dentistry, University of Salerno, Salerno, Italy

**Keywords:** sociomateriality, psychological development, human and non-human interactions, objects, contexts

## Introduction

The idea of sociomateriality mainly originates from the vast area of perspectives on psychological development related to empiricism. In simple terms, it could be said that sociomateriality stresses the contribution of individual and collective experience by putting more emphasis on the role that corporeity, physical contexts, and objects play in the development or emergence of psychological functions. Unfortunately, like any simplification, this one has objective limits. What makes it difficult to establish a unified framework to define sociomateriality, and above all to determine its relationship to psychological development, is first of all an epistemological question that is still the subject of a wide debate in several scientific areas, including philosophy (Searle, 2007) archaeology and material cultures (Malafouris, 2013), ergonomics (Geslin, 2017), anthropology and sociology (Latour, 2005), cognitive sciences (Clark, 2008), psychotherapy (Searles, 1960), developmental psychology (Moro and Rodríguez, 1998; Moro, 2016; Iannaccone et al., 2018) and learning itself (Engeström, 2015; Iannaccone, 2017; Cattaruzza et al., 2019). Within the limited extent of this introduction to the variegated Topic hosted by *Frontiers in Psychology*, we can identify the heart of the epistemological problem in two fundamental questions: (a) what are the boundaries of the mind with respect to corporeity and the context in which it operates? and (b) what could be the real contribution that artifacts give to the development of psychological functions, particularly learning?

Of course, these two problems not only have an abstract philosophical meaning, but also constitute a real methodological puzzle, because they question the notions of “object of analysis” and “unity of analysis.” To these important problems, researchers have given varied answers that are arranged along an axis with what we could define as “strong sociomateriality” on the one end and “weak sociomateriality” on the other. Concerning the explanations of psychological phenomena, this continuum depends substantially on the more or less decisive role that researchers assign to both the physical characteristics (materialities) of objects or contexts and to the communicative and semiotic interactions between humans and non-humans (social and cultural mediations). Even within this Topic, which is specifically dedicated to the role of objects in psychological development (affective, cognitive, and social), the contributions collected do not refer to a single notion of sociomateriality. On the positive side, these contributions present a rich landscape of theoretical and empirical positions requiring the reader to seriously reconsider sociomateriality in psychology. In summarizing the 14 contributions, we identified some common general aspects of the Topic that can help the reader organize his or her “journey”: Mental activities are not considered as decontextualized and isolated, but are interwoven in the interactions among individuals on one hand and the physical and social worlds on the other; and objects seem to actively contribute to typical and atypical psychological development (cognitive, affective, and social), influencing to several degrees the way that people experience the world. The contributions to the Topic are briefly presented below, organized according to their contribution to the issue of sociomateriality in psychology.

## Spaces of Activity and Objects

The function of the physical “spaces of activity” in children's learning clearly emerges in the case report of Barzanò et al. As in other similar research, the authors support the idea of “extended and situated” learning. This perspective integrates the formal context of the school with the informal spaces of other micro-contexts in which children and adults have their daily life experiences. Alternative spaces of activity influence the complexities of relationships and thus offer new opportunities to acquire alternative ways of exploring reality and learning.

Pinto et al. adopt sociomateriality as a theoretical lens to investigate how imitation acts to support the acquisition of the use of objects. Imitation, in the opinion of the authors, is a complex activity, involving several actors who interact to facilitate the understanding of various artifacts in different domains of knowledge and improve their interpretative flexibility between communities of practice.

Even at a much earlier stage of development, the importance of objects (among other variables) as elements of the newborn/parents' space of activity is partly highlighted in the research of Yamamoto et al. For example, the number of objects on the floor between infants and parents seems to modulate the eye contact with parents and activate some changes in the way that the child explores the surrounding context.

Granato analyses the neuropsychological effects of alcohol abuse, considering it as a real artifact both in its individual “use” as well as from an intergenerational perspective. Alcohol as a cultural artifact takes on different meanings depending on the individual and social practices that characterize its excessive intake.

Wang and Meltzoff describe a study with Chinese pre-school children. They report a number of theoretical views on imitation, assuming that it plays an important role in the early socio-cognitive development of children. One of the theoretical points of view refers to imitation as an act of social affiliation between the child and the adult, an activity also linked to socio-materiality. Through a series of tests of imitation with objects, researchers identified imitative activity as a key mechanism in the acquisition of culturally appropriate behaviors and conventions.

## Objects As Mediators of Some Complex Psychological Functions in Children With Autism Spectrum Disorders (ASD)

Manzi et al. adopt a sociomaterial approach to analyse interactions among children with ASD, adults and objects in a play setting. Systematic observations of object manipulation and communicative patterns displayed by children are conducted.

Assuming a perspective inspired by the Vygotskian concept of “psychological tools,” Manzi et al. highlight how objects can be considered (in some cases) as helpful mediators of communication, even in situations where interactions seem very problematic. The work also suggests a promising approach to supporting communicative patterns in children with ASD.

Marchetti et al. argue that motivated and shared actions directed toward an object can effectively mediate the child-adult relationship. According to this work, when face-to-face communication is challenging, as it is for example children with ASD, the presence of an object that encourages playful activities can expand the possibilities of communication by creating a triadic (child-adult) relationship.

Ponticorvo et al. explore the interweaving of cognitive and emotional dimensions with the nature of the materials used during the creative activities of children with ASD. The article shows how educational materials presented in both digital and physical form can effectively stimulate creativity.

Dimitrova's theoretical paper shows the importance of common background in communication between children and their parents in early childhood. Especially when the communicative setting refers to conventional uses of objects, common ground is an essential condition for a proper interpretation of the situation. In particular, a common background allows parents a well-adapted tailoring of their communicative response to the infant's developmental need.

## Interaction With Complex Objects: Human-Robot Interactions

The research of Di Dio et al. investigates the dynamics of trust (acquisition, loss and restoration) in children who interact with a humanoid robot or a human. The results show how in certain conditions, material artifacts can become referents with which to build relationships, modulated by the degree of anthropomorphization of the robotic agent.

The research of Manzi et al. shows how the degree of anthropomorphization affects the attribution of mental abilities to a robot by children of different ages. The results show that older children are more sensitive to the material characteristics of robots than younger children.

## Perception and Categorization of Objects

The study by Ishikawa et al. simulated the learning process of the child's gaze following: it emerged that the most feasible model is one in which communication signals influence the child's internal states. The model presented by the authors highlighted the importance of objects as a variable to be analyzed with respect to the communicative value of the adult's gaze.

The study of Taniguchi et al. analyses the way that infants classify objects at superordinate levels, considering the categories “living” or “not living.” The study shows how the categorization of living objects depends on linguistic development. According to the authors, this suggests different mechanisms in infants' categorization of living and non-living objects.

In further research, Taniguchi et al. investigate whether infants' decision to categorize objects depends on bottom-up and/or top-down processing (in relation to visual or verbal presentations). The authors also try to determine what visual information is required for quick and accurate categorization.

## Author Contributions

All authors listed have made a substantial, direct and intellectual contribution to the work, and approved it for publication.

## Conflict of Interest

The authors declare that the research was conducted in the absence of any commercial or financial relationships that could be construed as a potential conflict of interest.
